# Some Experiences with Extrauterine Pregnancy and Report of Cases1Read at the Buffalo Academy of Medicine, October 27, 1808.

**Published:** 1908-12

**Authors:** Herman E. Hayd

**Affiliations:** Buffalo, N. Y.


### BUFFALO MEDICAL JOURNAL.

VOL. LXIV. DECEMBER, 1908. No. 5

### ORIGINAL COMMUNICATIONS.

### Some Experiences with Extrauterine Pregnancy and Report of Cases[1]

By HERMAN E. HAYD, M. D., Buffalo, N. Y.

THE importance of this subject, together with its very frequent occurrence and the now universally accepted surgical treatment of the condition, has prompted me to write this paper, in which I shall try to deal, briefly and practically, with a few phases of the question and giving in its course, the conclusions which have been forced upon me as a result of an experience with seventy-three cases, in all stages of development, and perhaps with all possible complications and difficulties. After studying my records, they may be conveniently grouped into certain distinct and separate classes.

*First.*—Those in which rupture had not taken place.

*Second.*—Those in which sudden rupture occurred and a large vessel was opened, or a tubal abortion in which the bleeding continued freely into the peritoneal cavity, and where there was no tendency to localisation of the hemorrhage or the formation of an hematocele,—the so-called tragic or cataclysmic cases.

*Third.*—Cases in which rupture, partial or complete, had taken place,—including tubal abortion, partial and complete,—and where upon operation a large pelvic hematocele, more or less completely encysted, existed and where the ovum was so small that it either escaped observation or it was broken down in the blood clots which were in the sac contents. In this group can also be included those cases where the rupture took place on the under surface of the tube and the blood escaped into the folds of the broad ligament, producing a broad ligament hematocele, which, in my experience, is a rare variety and cannot often be diagnosticated until the abdomen is opened.

*Fourth.*—A group where rupture took place, but where the ovum continued to grow, say up to the fourth or fifth month, either within the peritoneal cavity or between the folds of the broad ligament, because it was not completely detached at the time of rupture, or because it made a new placental attachment.

1. Read at the Buffalo Academy of Medicine, October 27, 1808.

*Fifth.*—A class where the conception product broke down more or less completely and formed a pelvic abscess, or where its contents, pus, bones and debris, were being discharged either through the vagina, rectum, bladder or abdominal wall.

*Sixth.*—Where the fetus went on developing until it was viable or to term, and was then delivered by operation, or it died during labor and was finally removed as a lithopedion or other degenerated product.

Any form of pregnancy outside of the uterus is termed extra-uterine, and so far as we are interested, clinically anatomical distinctions are of little value; because it matters not whether the condition be one of tubal abortion or rupture in any part of the tube or cornu, the symptoms are practically the same and their relief must usually be brought about by surgical intervention.

The diagnosis of ectopic pregnancy before rupture takes place is seldom made because the cases do not come under observation early enough, and usually when a specimen is obtained early in the period of fecundation, it is because an operation was undertaken for some other condition, or where a tentative diagnosis was made with merely a suspicion of tubal pregnancy, because a slightly distended tube, whether by serum, blood or pus or an aberrant ovum, may give the same objective and subjective symptomatology. However, any tense, elastic and painful swelling of the tube, particularly when the swelling is confined to one side, and with a uterus reasonably movable, should always make the surgeon suspicious, especially if the period has been delayed, or if there has been present some irregular bleeding from the uterus. Pus in a tube, especially if slow in development, is usually associated with considerable exudate, and therefore more adhesions exist than are generally present with an early extrauterine pregnancy. Hydrosalpinx, in my experience, is comparatively rare, and hematoceles of the tube are most often the result of a slightly or completely detached extrauterine ovum, hence we are justified in making this general statement that any tense, elastic and painful swelling of the tube may be, and often is, an early extrauterine fetation, and should be most carefully watched, as an operation may become necessary at any moment.

In the examination of these women much care must be exercised, as the sac often ruptures as a result of the manipulations. This accident occurred to me once in one of my patients, whom I was examining in my office. She came, complaining of severe pain in the left side which made walking painful. During the examination I felt a painful, tense tumor in the left ovarian region, and while performing bimanual palpation, she was suddenly seized with an acute cramp-like pain; was nauseated and became faint. I removed her at once to the German Hospital, and opened her abdomen, which contained a large amount of free red fluid blood. The left tube had a rough, irregular tear in it near the fimbriated end, from which blood was flowing very freely. The ovum had escaped into the peritoneal cavity. The patient made a very rapid and excellent recovery.

A second patient,—which may be placed in either the first or second groups,—I saw in consultation with Dr. Gibson, to whom I referred the case, and as the history is exceedingly interesting though a very common one, I shall give it briefly:

Mrs. W., aged twenty-five, married two and one-half years. A fine, healthy English woman, who had always been well and regular, but at the last period she flowed rather profusely. She sent for a neighboring young doctor who thought she had a miscarriage, and proceeded at once to curet her, which he did without an anesthetic. Two days after this so-called curetment I saw her, and upon vaginal examination, discovered a small globular swelling in the left side, tense and painful. The uterus was tender, and pain was elicited upon gentle manipulation. I enjoined rest; applied poultices, and gave a little codeia to relieve the pain. She had a temperature of 100°. Upon the following day I saw her again, and turned the case over to Dr. Gibson, directing him to pay particular attention to a possible extrauterine fetation. As there was still some temperature, I felt that a few days should be taken to eliminate the swollen tube as a result of an infection with the traumatism consequent upon the curetage. Dr. Gibson reported that the patient was doing well, and that the husband had requested him to discontinue his visits, as he thought them unnecessary.

Four weeks after my first visit I was again called to the patient's house, as she was suffering great pain and had been in much distress for some days previous. Upon examination it was at once plainly evident that she was suffering from a large encysted hematocele, the result of a ruptured tubal pregnancy. I removed her to the German Hospital, and under chloroform, opened the culdesac and let out over a pint of black blood and blood clots of various sizes. The cavity was thoroughly irrigated with salt solution, and a piece of gauze was lightly packed into the vaginal cut for drainage. She did well for one week, the temperature remaining normal and then she began to complain of a good deal of pain, there being a constant elevation of temperature of a couple of degrees. On January 12, I opened her abdomen, and removed a large stinking mass, which was a ruptured tube with more or less organised broken-down blood clot. A guaze drain was placed low in the pelvis. She reacted nicely and did well, and on the thirteenth day, with normal temperature and normal pulse and bowels moving freely and good appetite, I left her in the care of one of my hospital associates, and took a trip East.

On the seventeenth day, trouble was noticed with her bowels, it being impossible to move them with gentle purgatives and injections, and she was always suffering from cramplike pains, but gas was still passing. I returned home February 3, and found her in a bad condition, crying with pain and much distended by gas, although a little gas occasionally passed naturally, but always with great pain. The bowels had not moved in six days, but there was no vomiting. I placed her at once upon the operating table, as it was evident a partial obstruction was rapidly becoming a complete one, and under chloroform anesthesia I reopened the abdominal wound and released a number of adhesions, separating and uncoiling a bad angle which existed in the pelvic sigmoid, and quickly closed the abdominal incision. She bore the operation well, and the bowels moved on the second day; she continued to do nicely, and made a most satisfactory recovery, and she is now a fine, strong, young woman.

If we review this case, we shall see that outside of the obstruction of the bowels, the history is quite common, and my notes record many cases which were mistaken for a miscarriage and were curetted by the attendant before the diagnosis of extrauterine pregnancy had been made. No doubt the slight temperature which existed and especially the pain upon examination, which I observed at my first visit, was increased by the curettage, but I felt positive that the swelling of the tube was a tubal fetation. However, she got better for a short time, and then no doubt a slight rupture of the tube took place, or the trophoblasts had bored and eaten their way through the tube wall and made small openings which permitted slight hemorrhages; these increased in amount and frequency until a large encysted hematocele resulted.

The second element of interest and of most importance to us, and what I trust this paper will provoke a good discussion upon, is what kind of surgery shall be employed and where shall the attack be made. There is not any question in my mind that free vaginal incision will cure many of these early cases, but I believe it is only good practice when the hematocele is recent and the blood not too strongly organised in the sac, and where there is no fetal product to be disposed of. If the condition has existed some time and the blood has become organised into more or less indissoluble bands, as this case of mine was, it is better practice to do a combined operation at the one sitting. First, incise the culdesac and empty out the sac contents, irrigate thoroughly and then open the abdomen and remove the whole mass. By first emptying the sac through the vagina, its walls collapse more or less; the bowel adhesions are more easily separated and the dangers of breaking the cyst wall and soiling the peritoneal cavity are reduced to a minimum. Vaginal drainage alone, when the case is not recent, implies a long convalescence, often a long run of fever, and for weeks a very dangerously sick woman, and often in the end a second operation to remove the unabsorbed mass with necessarily a high mortality.

In the second group, or tragic class, as Vineberg in a recent paper in the *Medical Record* discusses, are among the most terrible pictures the surgeon is called upon to care for. The symptoms are so sudden and terrific that a mistake in diagnosis should very rarely be made by any medical man with any experience. Some men have recently advocated a waiting policy, but I am not persuaded that it is good practice. On the contrary, in my view, an operation should be performed at once, and salt solution should be slowly and continuously injected into each breast throughout the whole operation, and to this solution adrenaline can often advantageously be added. One can never tell how much blood has been poured into the abdominal cavity, as pulse, hemaglobin count, or any other recent scientific deduction gives us practically no assistance. I have operated upon patients cold and pulseless, and have been surprised to see the pulse immediately come up just as soon as the abdominal cavity was opened and the intraabdominal pressure was relieved by the terrific gush of blood which came through the incision. The broken tube must be quickly found, and a clamp forceps applied and tied off with the greatest dispatch, and the abdomen closed with a few through and through silkworm sutures. The operating room should be very warm, and if possible the patient should lie on an electrically heated table, or any device that will keep up the body temperature should be continuously employed. Strychnia, digitalis and any recognised means of stimulation are to be used, and the patient is placed in bed, with the legs elevated. I have never had a patient in this condition die upon the table, and I cannot believe any have ever died by reason of the extra shock imposed by a rapid operation, who would not have died had no operation been performed, and the sense of well-being and comfort which comes to a surgeon who operates such a case and gets the woman off of the table is, indeed, very great. To stand by and anxiously look for a favorable turn, with all of its uncertainties before an operation is undertaken, is one of the most harrowing experiences I have ever subjected myself to; and, therefore, it has been my practice to operate at once every such case, no matter what condition I found the woman in, and my percentage of recoveries has been encouraging.

As a rule the technical difficulties of such operations are not great, as adhesions are not usually present, and no amount of diagnostic skill and acumen can foretell whether an operation will be difficult, and no tests or signs which are revealed by pulse and respiration,—our usual danger signals—are of much value here, because they do not signify how much free blood exists in the peritoneal cavity, or how much there still remains in the body bloodvessels; or how quickly the empty abdominal bloodvessels will drink up the fluid just as soon as the intraabdominal pressure and irritation are removed. In other words, the condition of extreme shock which these women are found in is not alone due to the mere loss of blood into their own belly cavities, but to the great shock to the sympathetic centers, the result of its quick and sudden accumulation in the peritoneal cavity, and every one of us has noticed when operating on these cases, how quickly the pulse comes up and the respirations improve when the black peritoneum is opened and the first sudden escape of fluid takes place. If the above premises and deductions be true, then it seems to me that we must come to an incontrovertible conclusion, that the waiting or delay policy of treating this class of cases is not good surgery. However, this, like many other surgical problems, must be solved by each surgeon according to his own light and experience and the conditions confronting him.

The diagnosis of the third and fourth groups, where hematocele exists and a detached fetus of recognisable size, can usually be easily established if sufficient time is taken to carefully work up the history of the case. The existence of a large swelling, the delayed or absent period, the irregular discharges of blood, pathognomonic in character, tarry, smeary and sticky, as Boldt pointed out in a recent paper, with shreds of decidual membrane, and sometimes even a perfect mold of the uterus, together with shooting pains in the rectum, and with that peculiar bearing down and forcing tenesmus seen in these cases, associated with many of the signs and symptoms of later pregnancy. Unfortunately, these cases are hurried to the operating table simply because a lump or tumor was felt upon vaginal examination, and a careful study of the symptoms was not systematically made. If the distended Douglas's pouch be opened, old blood or blood clots, or even pus, will freely flow away, or a digit—or other small bone—can often be pulled out of the mass, and if through this opening the examining finger be thrust, often much other valuable information can be obtained. Sometimes in these more advanced pregnancies, as has happened in a recent case with me, no fluid escaped through the culdesac incision; it was all absorbed and only the fetal and placental product remained. I successfully removed from this woman a four-month fetus which was dead and had partly undergone intrauterine, or better, intraperitoneal maceration, and yet the sac contained very little fluid contents.

The treatment of this group of cases I have already detailed, and I am sure my past five years' experience with the combined vaginal opening and copious irrigation, and then an immediate abdominal section, has lessened my previous mortality materially.

The fifth group simply presented the symptoms of pus in the pelvis, or pelvic abscess, and the history of a possible association with an extrauterine product was often not thought of. The long and painful illness, with the presence of a large and tender tumor, and the usual constitutional and local evidences of pus, made an operation imperative. In these cases it is also my practice to first evacuate the pus, if it can be reached through the Douglas's pouch, irrigate and then proceed at once to remove the remaining pathology by abdominal section, and drain below through the vagina with gauze or tube, together with properly placed guaze drains above, according to the indications of the case. If there exists above a dirty infected area of considerable size, I usually place a gauze wick or nest in each iliac fossa, removing them on the third to the sixth day.

I have never met an extrauterine fetation that has gone on living to term, nor have I operated upon such a case where a living baby existed; but I read at the meeting of the American Association of Obstetricians and Gynecologists, in Detroit, a paper which dealt with a most interesting case of a lithopedion which I successfully removed from a woman sixty-seven years of age, and which had existed for thirty-two years. And that paper, with photographs, was printed in the December number, 1907, *American Journal of Obstetrics*, and I shall pass around the original photographs of the specimen, which is now preserved in the Pathological Museum of the Medical Department of the University of Buffalo.

493 Delaware Avenue.

